# Reduced responsiveness of the reward system is associated with tolerance to cannabis impairment in chronic users

**DOI:** 10.1111/adb.12870

**Published:** 2019-12-22

**Authors:** Natasha L. Mason, Eef L. Theunissen, Nadia R.P.W. Hutten, Desmond H.Y. Tse, Stefan W. Toennes, Jacobus F.A. Jansen, Peter Stiers, Johannes G. Ramaekers

**Affiliations:** ^1^ Department of Neuropsychology and Psychopharmacology, Faculty of Psychology and Neuroscience Maastricht University Maastricht The Netherlands; ^2^ Institute of Legal Medicine University of Frankfurt Frankfurt/Main Germany; ^3^ Department of Radiology and Nuclear Medicine Maastricht University Medical Center+ (MUMC+) Maastricht The Netherlands; ^4^ School for Mental Health and Neuroscience Maastricht University Medical Center Maastricht The Netherlands

**Keywords:** cannabis, glutamate, tolerance

## Abstract

Cannabis is the most commonly used illicit drug in the world. However, because of a changing legal landscape and rising interest in therapeutic utility, there is an increasing trend in (long‐term) use and possibly cannabis impairment. Importantly, a growing body of evidence suggests that regular cannabis users develop tolerance to the impairing, as well as the rewarding, effects of the drug. However, the neuroadaptations that may underlie cannabis tolerance remain unclear. Therefore, this double‐blind, randomized, placebo‐controlled, cross‐over study assessed the acute influence of cannabis on the brain and behavioral outcomes in two distinct cannabis user groups. Twelve occasional and 12 chronic cannabis users received acute doses of cannabis (300‐μg/kg delta‐9‐tetrahydrocannabinol) and placebo and underwent ultrahigh field functional magnetic resonance imaging and magnetic resonance spectroscopy. In occasional users, cannabis induced significant neurometabolic alterations in reward circuitry, namely, decrements in functional connectivity and increments in striatal glutamate concentrations, which were associated with increases in subjective high and decreases in performance on a sustained attention task. Such changes were absent in chronic users. The finding that cannabis altered circuitry and distorted behavior in occasional, but not chronic users, suggests reduced responsiveness of the reward circuitry to cannabis intoxication in chronic users. Taken together, the results suggest a pharmacodynamic mechanism for the development of tolerance to cannabis impairment, of which is important to understand in the context of the long‐term therapeutic use of cannabis‐based medications, as well as in the context of public health and safety of cannabis use when performing day‐to‐day operations.

## Graphical abstract

Using an ultra‐high field multimodal brain imaging approach, we showed that in occasional cannabis users, acute THC administration increased striatal glutamate and decreased functional connectivity in the reward system, which were associated with increases in subjective high and decreases in sustained attention. Such changes were absent in chronic cannabis users. Findings suggest the development of neuroadaptations in reward circuitry after excessive use of cannabis, which may underlie behavioral tolerance to the drug.

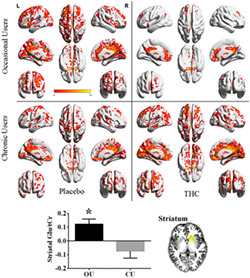

## 1 INTRODUCTION

Cannabis is the most commonly used illicit drug in the world, with 4% of the global population reportedly using it.^1^ However, because of a changing legal landscape and rising interest in therapeutic utility, there is an increasing trend in (long‐term) use.^1^, ^2^ Importantly, a growing body of evidence suggests that the acute effects of cannabis are less prominent in regular cannabis users,^3^ suggesting development of tolerance to the impairing, as well as the rewarding, effects of the drug. Nonetheless, the neurobiological mechanisms underlying cannabis tolerance are unknown.

Accumulating evidence suggests that the main psychoactive component of cannabis (delta‐9‐tetrahydrocannabinol THC]) binds to cannabinoid (CB1) receptors located on GABAergic and glutamatergic neurons distributed throughout the brain, with high densities found in limbic‐reward structures.^4^ Subsequently, THC has been found to acutely activate the reward circuitry, increasing dopamine^5^, ^6^, ^7^, ^8^, ^9^ and glutamate^10^, ^11^ concentration levels in key brain areas including the striatum, nucleus accumbens (NAc), and prefrontal cortex (PFC), a pattern implicated in both the rewarding and impairing effects of drugs of abuse.^12^, ^13^

Accordingly, studies with *chronic* cannabis users have found alterations in dopaminergic function in striatal areas,^14^, ^15^, ^16^ as well as decreases in glutamate concentrations in the basal ganglia^17^, ^18^ and anterior cingulate cortex (ACC).^19^, ^20^ Furthermore, repeated use of cannabis has been associated with structural changes in frontal areas, as evinced by decreased gray matter volume,^21^, ^22^, ^23^ and abnormal concentrations of metabolites including N‐acetylaspartate (NAA), myo‐inositol (mI), and choline‐containing compounds (Cho), biochemical markers of neuronal integrity and glial activation.^18^, ^19^, ^24^, ^25^, ^26^, ^27^, ^28^ Taken together, these studies provide evidence that repeated cannabis exposure may lead to alterations in neurotransmission and neuronal health, which underlie the diminished cognitive and behavioral response associated with acute cannabis tolerance. However, to date, the neuroadaptations that may underlie cannabis tolerance have not been systematically assessed.

Therefore, the aim of the present double‐blind, placebo‐controlled study was twofold. The first goal was to assess acute influence of cannabis (300‐μg/kg THC) in two different cannabis using groups, namely, occasional and chronic users, on the brain and behavioral outcomes previously found to be affected by cannabis. Ultrahigh field (7T) proton magnetic resonance spectroscopy (MRS) was used to assess glutamate, GABA, NAA, Cho, and mI levels in the striatum and ACC. Resting‐state functional magnetic resonance imaging (fMRI) data were acquired to determine functional connectivity (FC) between the regions of interest (ROI) in the NAc and remote cortical areas, as an indirect measure of dopaminergic stimulation.^10^, ^29^, ^30^ Furthermore a priori ROI‐to‐ROI analysis assessed differences in connectivity strength between areas of the reward circuit. Finally, subjective high and sustained attention, two outcome variables shown to be modulated by (acute) cannabis exposure,^10^, ^31^, ^32^ were assessed.

The second goal was exploratory, to evaluate long‐term effects of repeated cannabis exposure, by comparing the placebo condition of each group. Overall, we hypothesized that THC would induce behavioral, functional, and metabolic changes in occasional, but not chronic users, indicative of (neuroadaptive) tolerance. Furthermore, on the basis of previous studies with chronic users, we hypothesized that during placebo, chronic users would show decreased concentrations of metabolites compared with occasional users.

## 2 MATERIALS AND METHODS

A detailed description of the methodology is provided in the Supporting Information and briefly summarized here.

The study was conducted according to a double‐blind, placebo‐controlled, mixed cross‐over design. Twelve occasional and 12 chronic users (male, N = 14) received placebo and 300‐μg/kg THC (Bedrobinol; 13.5% THC) on separate days, separated by a minimum wash‐out period of 7 days for occasional users. Sample size was based off of power calculations and previous studies assessing reward system activation during acute THC intoxication.^10^, ^30^

This study was conducted according to the code of ethics on human experimentation established by the declaration of Helsinki (1964) and amended in Fortaleza (Brazil, October 2013) and in accordance with the Medical Research Involving Human Subjects Act (WMO) and was approved by the Academic Hospital and University's Medical Ethics committee. All participants were fully informed of all procedures, possible adverse reactions, legal rights and responsibilities, expected benefits, and their right for voluntary termination without consequences. All participants gave their informed consent, in writing.

### 2.1 Image acquisition

All participants underwent a functional MRI and single‐voxel MRS. Images were acquired on a MAGNETOM 7T MR scanner.

Spectroscopic voxels were placed in the ACC (voxel size = 25 × 20 × 17 mm^3^) and the right striatum (voxel size = 20 × 20 × 20 mm^3^). Spectra were acquired with stimulated echo acquisition mode^33^ sequence (TE = 6.0 ms, TR = 5.0 s, 64 averages). Outcome measures for MRS were concentration ratios of glutamate, GABA, NAA, mI, and Cho to total Creatine (tCr, Creatine + Phospho‐Creatine).

Additionally, 258 whole‐brain EPI volumes were acquired at rest (TR = 1400 ms; TE = 21 ms; flip angle = 60°; oblique acquisition orientation; interleaved slice acquisition; 72 slices; slice thickness = 1.5 mm; voxel size = 1.5 × 1.5 × 1.5 mm). During scanning, participants were shown a black cross on a white background and were instructed to focus on the cross while attempting to clear their mind.

### 2.2 Processing of imaging data

Spectroscopy data were analyzed with LCModel version 6.3‐1H.

FC data were produced with the MATLAB toolbox DPARSF.^34^ In order to indirectly assess dopamine neurotransmission, two spheres (4‐mm radius) were created that were located (in MNI space) in the left and right NAc. Average time courses were obtained for each sphere separately, and correlational analysis was performed voxel wise to generate FC maps for each sphere.

Furthermore, as we were interested in FC within the reward circuit, ROI‐to‐ROI FC was computed according to the same aforementioned procedure, between areas including NAc, medial dorsal nucleus (MDN), ventral pallidum (VP), and midcingulate area (MC). Fisher correlation coefficient maps were created between the NAc and MDN, NAc and VP, MDN and VP, MDN and MC, and MC and NAc.

### 2.3 Subjective and behavioral measures

Sustained attention was assessed via the psychomotor vigilance task (PVT), a reaction‐time task that measures the speed with which participant responds to a visual stimulus.^35^ The outcome measures of the task are response speed (mean reaction time) and number of attentional lapses (reaction time >500 ms).

Participants also rated their subjective high on visual analog scales (10 cm) on four consecutive time points after treatment administration, on a scale between 0 (*not high at all*) and 10 (*extremely high*).

### 2.4 Pharmacokinetic measures

Blood samples (8 mL) to determine cannabinoid concentrations (THC and metabolites OH‐THC and THC‐COOH) were taken at baseline, 10, 30, 50, and 70 minutes postadministration, and analyzed according to a standardized procedure.^10^

## 3 STATISTICAL ANALYSIS

### 3.1 Subjective high, sustained attention, and metabolite concentrations

A mixed‐model analysis was performed consisting of the within‐subject factors treatment (THC and placebo), time after smoking (two levels), and the between‐subject factor of group (occasional or chronic) (SPSS Version 24; SPSS Inc, Chicago, IL). Because of main effect of Treatment or interaction of Treatment X Group, a second analysis was performed for each group, with treatment and time as within‐subject factors. The alpha criterion level of significance was set at *P* = .05. Because of a violation of the assumption of normality, the data for the number of lapses and mean reaction time were log transformed.

### 3.2 fMRI data

FC data (ie, correlation coefficient maps for each individual in each treatment condition at each time point) were analyzed in a GLM model in SPM 12.

For the reward circuit, ROI‐to‐ROI analysis of Fisher correlation coefficient values was conducted in IBM SPSS statistics 24. For each group, a repeated measures analysis was conducted consisting of the within‐subject factor treatment (THC and placebo).

### 3.3 Correlation analysis

Correlation analyses were conducted to further investigate the relationship between cannabis‐induced changes in the brain and behavior. Correlation input included average treatment change values of (a) behavioral outcomes (subjective high and number of lapses), (b) MRS concentration levels, (c) ROI‐to‐ROI Fisher correlation values, and (d) mean voxel activation of SPM identified clusters from a voxel‐wise correlation analysis between NAc FC and behavioral outcomes. Pearson correlations were performed in SPSS.

### 3.4 Exploratory analysis

In order to assess long‐term effects of repeated cannabis exposure, separate analyses of covariance were carried out for average (placebo) neurometabolite concentrations, behavioral measures, and FC in the reward circuit, as the dependent variables and group (occasional vs chronic user) as the fixed factor. Serum THC, 11‐OH‐THC, and THC‐COOH levels were entered as covariates, because of significant differences between groups.

## 4 RESULTS

### 4.1 Demographic characteristics

Occasional users (n = 12) and chronic users (n = 12) did not differ with respect to gender distribution, age, history of cannabis use, or consumption of alcohol, caffeine, nicotine, or other drugs (Table S1). As expected, chronic users reported using significantly more cannabis per week than occasional users.

### 4.2 THC concentrations in serum

Mean (SE) concentrations of THC, 11‐OH‐THC, and THC‐COOH in serum are given in Table S2. As expected from previous experience,^36^ chronic users not only exhibited significantly higher THC‐COOH concentrations but also reached significantly higher THC levels from the same dose regimen than occasional users.

### 4.3 Subjective and cognitive effects

Mixed‐model analyses of variance (ANOVA) [Treatment (THC vs placebo) * Time (timepoint 1 vs timepoint 2) * Group (occasional vs chronic users)] yielded a significant main effect of Treatment [*F*(1,21) = 49.682, *P* < .0001, *ηp* ^2^ = .703] and Time [*F*(3,63) = 42.269, *P* < .0001, *ηp* ^2^ = .668] and a significant interaction of Treatment * Group [*F*(1,21) = 5.023, *P* = .036, *ηp* ^2^ = .193] and Treatment * Time [*F*(363) = 17.346, *P* < .0001, *ηp* ^2^ = .452] on subjective high, indicating that THC increased feelings of intoxication in both groups but to a larger degree in occasional users (Figure S1A).

Analysis yielded a significant main effect of Treatment [*F*(1,20) = 8.057, *P* = .010, *ηp* ^2^ = .287] and Time [*F*(1,20) = 7.620, *P* = .012, *ηp* ^2^ = .276] and a significant interaction of Time * Group [*F*(1,20) = 8.085, *P* = .010, *ηp* ^2^ = .288] on mean reaction time of the PVT. Further analysis revealed that mean reaction time was significantly increased by THC in occasional users [*F*(1,10) = 5.226, *P* = .045, *ηp* ^2^ = .343] but not in chronic users (*P* > .1) (Figure S1B).

Analysis yielded a significant main effect of Treatment [*F*(1,21) = 7.793, *P* = .011, *ηp* ^2^ = .271] and a significant interaction of Time * Group [*F*(1,21) = 4.313, *P* = .050, *ηp* ^2^ = .170] on number of attentional lapses during the PVT. Further analysis revealed that number of attentional lapses was significantly increased by THC in occasional users [*F*(1,10) = 5.286, *P* = .044, *ηp* ^2^ = .346] but not in chronic users (*P* > .1) (Figure S1C).

## 5 METABOLITE CONCENTRATIONS

### 5.1 Striatum

**FIGURE 1:**
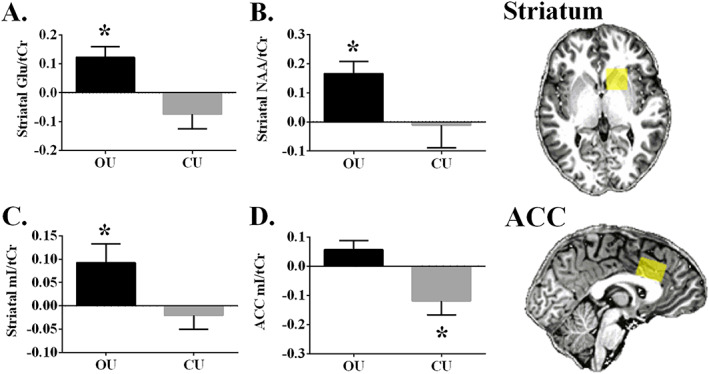
Occasional and chronic users mean (SE) metabolite concentration levels, averaged over both timepoints for both treatments [average (THC timepoint 1 − Placebo timepoint 2; THC timepoint 2 − Placebo timepoint 2)]. A, Striatal glutamate. B, Striatal total n‐acetyl‐aspartate. C, Striatal myoinositol. D, Anterior cingulate cortex myoinositol. OU, occasional user; CU, chronic user; *within group analysis, *P* < .05

Mixed‐model ANOVA yielded a significant interaction of Treatment * Time * Group [*F*(1,21) = 8.779, *P* = .007, *ηp* ^2^ = .295] on glutamate/tCr (Glu) concentration levels. Further analysis revealed that concentration levels were significantly increased by THC in occasional users [*F*(1,11) = 11.506, *P* = .006, *ηp* ^2^ = .511] but not in chronic users (*P* > .1) (Figure 1A).

Analysis yielded a significant interaction of Treatment * Time * Group [*F*(1,19) = 6.546, *P* = .019, *ηp* ^2^ = .256] and a significant main effect of Treatment [*F*(1,19) = 4.670, *P* = .044, *ηp* ^2^ = .197] on NAA + NAAG/tCr (NAA) concentration levels. Further analysis revealed that concentration levels were significantly increased by THC in occasional users [*F*(1,11) = 15.345, *P* = .002, *ηp* ^2^ = .582] but not in chronic users (*P* > .1) (Figure 1B).

Analysis yielded a trending interaction of Treatment * Group [*F*(1,20) = 4.241, *P* = .053, *ηp* ^2^ = .175] on mI/tCr (mI) concentration levels. Further analysis revealed that concentration levels were significantly increased by THC in occasional users [*F*(1,11) = 5.176, *P* = .044, *ηp* ^2^ = .320] but not in the chronic users (*P* > .1) (Figure 1C).

### 5.2 Anterior cingulate cortex

Mixed‐model ANOVA yielded a significant interaction of Treatment * Group [*F*(1,18) = 7.450, *P* = .014, *ηp* ^2^ = .293] on mI concentration levels. Further analysis revealed that concentration levels were significantly decreased by THC in chronic users [*F*(1,9) = 5.935, *P* = .038, *ηp* ^2^ = .397] but not in occasional users (*P* > .1) (Figure 1D)].

Other metabolites did not reach significance (see Table S3 for mean metabolite concentrations).

### 5.3 FC with the NAc

**FIGURE 2:**
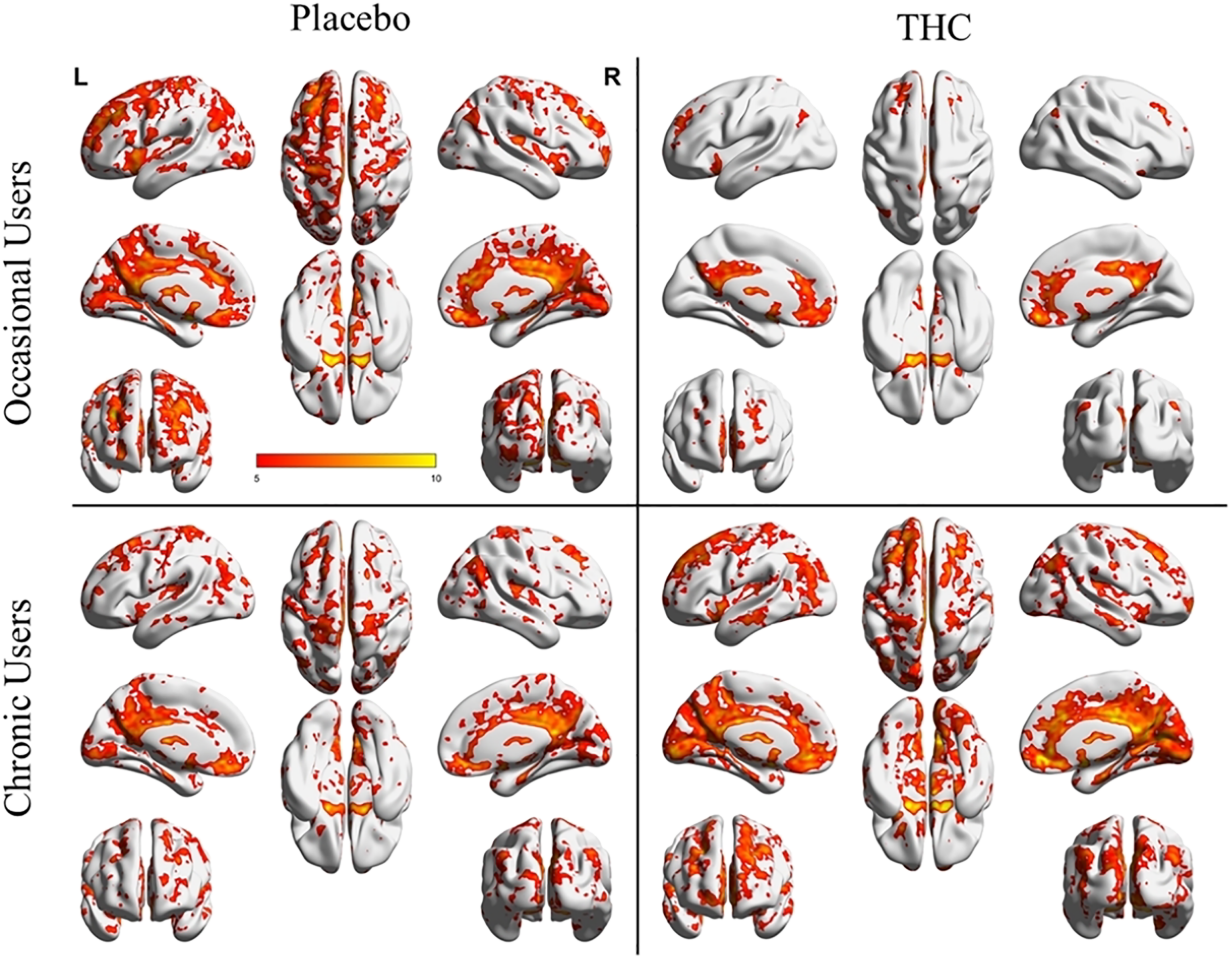
NAcc‐related functional connectivity in the left hemispheres. Shown are thresholded *Z*‐score maps of functional connectivity for each group and each condition

The contrast drug vs placebo (placebo > THC) resulted in reduced FC with the NAc seeds in both hemispheres in occasional users, whereas no change was found in chronic users (Figure 2). Reductions in FC were prominent in broad areas of the frontal, temporal, parietal, and occipital lobes, a pattern typical of an increase in dopaminergic neurotransmission (Table S4). No significant differences in activation were found for the inverse comparison (THC > placebo) in either group. Furthermore, no significant differences were seen between the left and right NAc seed, so only left NAc seed results are shown.

### 5.4 FC in the reward circuit

**FIGURE 3:**
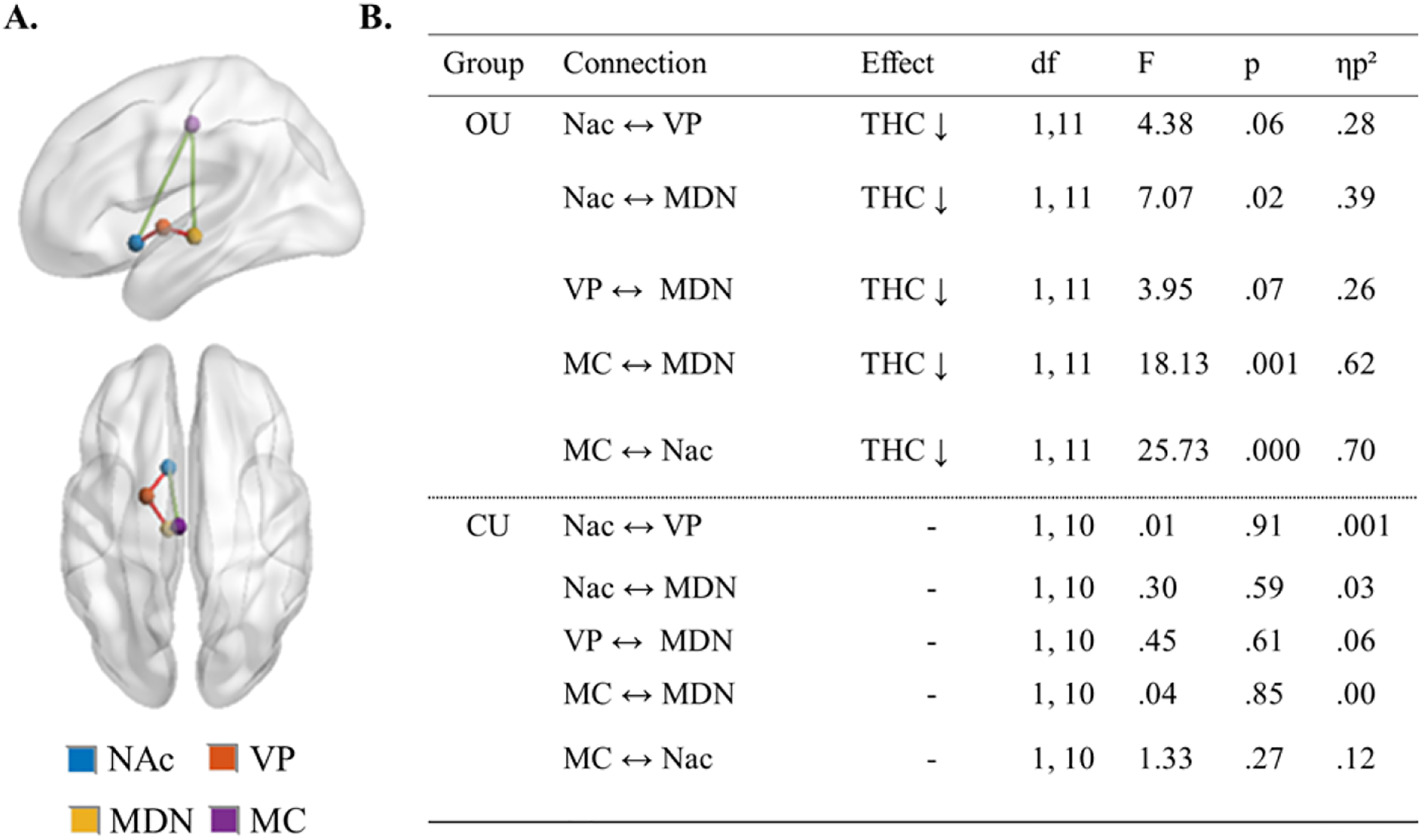
ROI‐to‐ROI FC analysis results. A, Four nodes of the reward circuit, where ROIs were placed. Red lines pertain to GABAergic pathways, whereas green lines pertain to glutamatergic pathways. NAc, nucleus accumbens; VP, ventral pallidum; MDN, medial dorsal nucleus; MC, midcingulate area. B) Results of repeated measures analysis (THC vs placebo), separated by group. OU, occasional user; CU, chronic user

Results of the ROI‐to‐ROI FC analysis are displayed in (Figure 3). ROIs were chosen because they are established structures of the reward circuitry, a cortico‐subcortical network connected via glutamatergic and GABAergic projections between the NAc, VP, MDN, and the PFC^37^, ^38^, ^39^, ^40^ (Figure 3). NAc, VP, and MDN seeds were chosen a priori, whereas the prefrontal seed was chosen based on the previous FC analysis, which indicated significant treatment (placebo > THC) induced changes in the MC. Mixed‐model ANOVA revealed that cannabis decreased FC between the ROIs in occasional users, whereas no significant treatment effect was seen in chronic users.

### 5.5 Relationship between cannabis‐induced changes in brain and behavior

Analysis revealed a significant positive correlation in occasional users (*r* = .641) between treatment‐induced changes in striatal NAA and number of attentional lapses and a significant negative correlation (*r* = −.618) between treatment‐induced changes in striatal mI and subjective high. For an overview of all correlational analyses, see Table S7. A further voxel‐wise correlation analysis between NAc FC whole‐brain correlation coefficient maps and behavioral outcomes was performed, revealing significant positive correlations (all *r* > .6) in FC between the NAc and cortical brain areas and behavioral outcomes (Table S8).

### 5.6 Exploratory analysis

No significant difference between groups was found in any of the variables of interest, when controlling for THC and THC‐metabolite concentrations in blood (*P* > .1).

## 6 DISCUSSION

The present study demonstrates the first attempt to assess cannabis‐induced neuroadaptations in the reward system, which may underlie behavioral cannabis tolerance. Using an ultrahigh field multimodal brain imaging approach, we showed that in occasional users, cannabis induced significant neurometabolic alterations in the reward circuitry, namely, decrements in FC and increments in striatal glutamate, which were associated with increases in subjective high and decreases in performance on a sustained attention task. Such changes were absent in chronic users. The finding that cannabis altered reward circuitry and distorted behavior in occasional, but not chronic users, suggests the development of neuroadaptations in the reward circuitry after excessive use of cannabis that reduces the circuitry and behavioral response to acute cannabis impairment.

The present study found that, in occasional users, cannabis decreased coupling between BOLD responses in keys areas of the reward system, a fronto‐subcortical network of brain structures that are connected via dopaminergic, GABAergic, and glutamatergic neurotransmission (ie, NAc, VTA, and VP), motivation and salience attribution (ie, medial orbital frontal cortex), executive and inhibitory control (ie, ACC), and conditioning and memory (ie, amygdala, medial orbital frontal cortex, and hippocampus).^41^, ^42^ Importantly, decrements in FC between the NAc and regions such as the thalamus and frontal cortex have been suggested to reflect increases in dopaminergic neurotransmission throughout the circuit.^10^, ^29^, ^30^ Specifically, the NAc receives dopaminergic input from the VTA, which is under inhibitory control of GABA interneurons on which presynaptic CB1 receptors are located. Stimulation of CB1 receptors by THC disinhibits the VTA, which in turn increases dopamine levels in the NAc.^43^ Subsequently, the increase in striatal dopamine level output from the NAc decreases the GABAergic inhibitory tone to the thalamus, which is reflected in decreased FC, as seen in this study.

Furthermore, this process of disinhibition has been suggested to lead to increased glutamatergic signaling to the PFC, subsequently to the VTA, and back to the NAc.^30^ Accordingly, MRS in occasional users showed cannabis increased striatal glutamate concentration levels, a finding that is compatible with previous human studies that have found acute increases,^10^, ^11^ as well as chronic decreases^18^ of striatal glutamate after exposure to THC. Furthermore, it has previously been demonstrated that cannabis‐induced changes in striatal glutamate levels correlate strongly to cannabis‐induced alterations of FC within the fronto‐subcortical circuit.^10^ Thus, it could be hypothesized that stimulatory glutamatergic input from the PFC to the NAc synergizes with cannabis‐induced increases in dopaminergic input from the VTA to the NAc and further strengthens the disinhibition of thalamic signaling in the fronto‐subcortical circuitry. However, MRS did not show altered ACC glutamate concentrations in the OU group, as would be hypothesized. Indeed previous results regarding the effects of cannabis on glutamate in the ACC have been mixed, with studies by one group finding reductions of glutamate‐related metabolite concentrations in cannabis users,^19^, ^20^ whereas no alterations were found in a subsequent chronic^28^ or acute^10^ study. Nonetheless, previous work utilizing microdialysis found THC increased extracellular glutamate in the rat PFC,^9^ supporting the hypothesis that THC disrupts glutamatergic signaling in frontal areas; however, this effect may not be localized to the ACC. Thus, future studies should assess the impact THC has on other areas in the frontal cortex, like the medial orbitofrontal cortex.

The present study also demonstrated that cannabis increased feelings of subjective high and decreased performance on a sustained attention task in occasional users, outcome variables previously found to be affected by cannabis.^10^, ^31^, ^32^ Furthermore, behavioral outcomes correlated with striatal FC to other areas of the reward circuit. Accordingly, cannabis‐induced changes in striatal glutamate and striatal FC have been significantly associated with decrements in cognitive function and impulse control,^30^ as well as increases in subjective high^10^, ^30^ and experience of psychotomimetic symptoms.^11^ Taken together, the findings suggest that the impact of cannabis on neural activity within the reward circuit may underlie multiple behavioral changes observed after acute cannabis exposure.

In line with this, chronic users demonstrated an absence of cannabis‐induced stimulation of the reward circuit, as well as mitigation of behavioral alterations. Specifically, no changes were seen in either FC between areas of the reward circuitry, or glutamate concentration levels, when comparing cannabis to placebo. Furthermore, sustained attention performance did not significantly differ between treatment conditions. However, chronic users reported significantly increased levels of subjective high after cannabis relative to placebo, although the change in high was to a lesser extent than in the occasional users, as expected.^31^ Taken together, findings suggest that chronic cannabis users exhibit pharmacodynamic tolerance to the effects of cannabis.

The mechanisms by which neurobiological tolerance to the acute effects of THC develops have yet to be fully elucidated. Animal and human research generally supports the notion of CB1 receptors downregulation and desensitization in cortical and subcortical regions after repeated exposure to cannabis.^44^, ^45^, ^46^, ^47^, ^48^, ^49^, ^50^, ^51^ Although studies have reported global reduction in CB1 receptor availability in chronic cannabis users, it has been suggested that neuroadaptive changes take place in a time‐ and region‐specific manner,^52^ with regional analysis demonstrating significant CB1 receptor decrements in areas such as the ACC and NAc.^50^ Thus, the absence of change in FC of the NAc with other parts of the reward circuit could suggest that downregulation of CB1 receptors mitigates the impact of acute cannabis intoxication on neural activity within fronto‐subcortical circuits and associated behavioral outcomes, thus demonstrating the prime mechanisms underlying the development of tolerance in this circuit. However, to further explore the association between CB1 receptor availability and development of tolerance, future studies should assess CB1 receptor availability during acute intoxication.

In order to assess potential long‐term effects of repeated cannabis exposure, analyses were performed comparing the placebo conditions between chronic and occasional users on ROI‐to‐ROI FC within the reward circuit, metabolite concentration levels, and performance on the sustained attention task. As chronic users had significantly higher baseline serum THC and THC‐metabolite levels than occasional users, these values were added as covariates. When controlling for serum concentration levels, no differences were seen between groups on any of the outcome variables. Absence of group differences is in line with previous PET studies, which have found CB1 receptor availability normalization in cannabis‐dependent users after as little as 2 days of monitored abstinence,^49^, ^51^ as well as neuropsychological data suggesting reversible cognitive deficits, modulated more by recent exposure than by cumulative lifetime use.^53^ However, the literature on long‐lasting effects of cannabis use is mixed, with studies also reporting long‐term changes on brain structure,^22^, ^23^ neurometabolite levels,^54^ and neurocognitive functioning.^55^ Importantly, results have been found to be region and domain specific and influenced by factors such as frequency and age of onset of use, potentially explaining the variance in reported outcomes. In order to further assess chronic effects of repeated cannabis exposure on the reward system and associated behavior, future studies should employ a larger sample size and take such factors into account.

Finally, MRS demonstrated cannabis‐induced changes in neurometabolites previously found to be altered in cannabis users,^54^ namely, NAA and mI. NAA and mI, as well as glutamate, are markers of glial and neuronal activation.^56^, ^57^ Our data demonstrate that cannabis acutely increases these metabolites in the striatum in occasional users. Treatment‐induced changes in NAA and mI also correlated with behavioral changes. Specifically, it was found that cannabis‐induced changes in mI in the striatum negatively correlated with cannabis‐induced changes in subjective high. Interestingly, elevations in mI and glutamate have been found in individuals with first‐episode psychosis and were found to correlate with subjective reports of grandiosity.^58^ Furthermore, in the present study, it was found that cannabis‐induced changes in NAA positively correlated with cannabis‐induced changes in sustained attention performance. Similarly, altered levels of NAA and NAAG have been reported in patients with schizophrenia, albeit in the ACC, with NAA levels correlating with attention performance.^59^ Taken together, results suggest that cannabis increases glial and neuronal activation in the striatum, which is associated with changes in subjective state and behavioral performance. Importantly, these changes were not seen in the chronic users, further demonstrating pharmacodynamic tolerance. However, cannabis decreased mI in the ACC in chronic, but not occasional, users. The finding of acute decrease in ACC mI is compatible with previous studies that found decreased mI in the ACC,^19^, ^20^ as well as throughout the brain,^26^, ^27^ and has been suggested to reflect cannabis‐related immunosuppression.^54^

In summary, our study provides previously unidentified evidence to suggest that a reduced responsiveness of the reward circuitry underlies a blunted pharmacodynamic response to an acute cannabis challenge in chronic users. Understanding the neuroadaptive basis of tolerance is important in the context of the therapeutic use of cannabis‐based medications, as well as in the context of public health and safety of cannabis use when performing day‐to‐day operations.

## CONFLICT OF INTEREST

The authors declare no conflict of interest.

## Supporting information



Data S1 Supporting Information
